# The burden of acute malnutrition among children under five in conflict-afflicted Gaza strip: prevalence and associated factors

**DOI:** 10.3389/fnut.2024.1478485

**Published:** 2024-11-27

**Authors:** Ahmed Albelbeisi, Kate Zinszer, Abdel Hamid El Bilbeisi, Samer Abuzerr

**Affiliations:** ^1^College of Health Professions, Israa University, Gaza City, Palestine; ^2^Department of Social and Preventive Medicine, School of Public Health, University of Montreal, Montréal, QC, Canada; ^3^Department of Nutrition, School of Medicine and Health Sciences, University of Palestine, Gaza City, Palestine; ^4^Department of Medical Sciences, University College of Science and Technology, Khan Younis, Palestine

**Keywords:** severe acute malnutrition (SAM), moderate acute malnutrition (MAM), nutritional status, mid-upper arm circumference (MUAC), child health, socio-demographic determinants, humanitarian crisis, health interventions

## Abstract

**Background:**

This study aims to assess the prevalence and associated factors of Severe Acute Malnutrition (SAM) and Moderate Acute Malnutrition (MAM) among children in this region during a period of conflict.

**Methods:**

A cross-sectional study was conducted with a sample of 1,200 children under 5 years old, selected through stratified random sampling from three governorates in the Gaza Strip. Data were collected using structured interviews and physical examinations, including Mid-Upper Arm Circumference (MUAC) measurements to determine nutritional status based on established cut-off points. Statistical analyses were performed using IBM-SPSS, version 26.

**Results:**

Among the 1,200 children screened, 605 (50.8%) were males and 595 (49.2%) were females. The age distribution of the children screened showed that 48.4% (*n* = 581) fall within the range of 6 months to <24 months category and 51.6% (*n* = 619) fall within the range of 24–60 months. Overall, 58.3% of children live in schools, 26.6% live in houses, and 15.1% reside in tents. The overall prevalence of malnutrition across all regions was 16.6%, including 6.7% with SAM and 9.7% with MAM. The prevalence of SAM is highest in North Gaza and South Gaza (both at 6.8%) and slightly lower in the Middle Zone (6.5%). For MAM, the highest prevalence is in South Gaza (11.5%), with Middle Zone showing the lowest rate (7.3%). Logistic regression analysis indicated that children living in houses had a higher likelihood of SAM (OR = 2.36; 95% CI = 1.39–3.99) and MAM (OR = 1.44; 95% CI = 1.13–1.84), and children living in schools had a higher likelihood of SAM (OR = 2.79; 95% CI = 1.35–5.74) and MAM (OR = 2.08; 95% CI = 1.14–3.80) compared to those in tents. Notably, children residing in North Gaza were significantly more likely to experience SAM (OR = 13.2; 95% CI = 6.23–27.95) and MAM (OR = 10.51; 95% CI = 5.74–19.3) compared to those in South Gaza.

**Conclusion:**

The study highlights a high prevalence of malnutrition among children under five in the Gaza Strip, particularly among those living in houses, shelters and in the North Gaza governorate. These findings underscore the urgent need for targeted nutritional interventions and support for affected families in conflict zones.

## Introduction

Malnutrition remains a significant global health issue, particularly affecting children under the age of five (1). This problem is further exacerbated in conflict zones, where access to essential resources such as food, clean water, and healthcare is severely compromised (2–4). The Gaza Strip, a region plagued by prolonged political and military conflict, presents a stark example of how war and instability can lead to severe health crises among vulnerable populations, especially young children (5, 6).

In conflict-affected areas like Gaza, the disruption of normal living conditions and services can lead to increased rates of malnutrition (7, 8). Families are often displaced, and infrastructure, including healthcare facilities and food supply chains, is frequently damaged or destroyed (9, 10). As a result, children under 5 years old are at an elevated risk of developing Severe Acute Malnutrition (SAM) and Moderate Acute Malnutrition (MAM) (11, 12). These conditions not only jeopardize their immediate health and survival but also have long-term impacts on their physical and cognitive development (13, 14). Mid-Upper Arm Circumference (MUAC) is particularly valuable in conflict settings like Gaza due to its simplicity, ease of use, and effectiveness in identifying acute malnutrition in resource-limited environments where more comprehensive assessments may not be feasible (15).

Previous studies have highlighted the dire nutritional status of children in the Gaza Strip, noting a high prevalence of malnutrition and its associated risk factors (16–18). Some of these studies have utilized MUAC as a nutritional assessment tool, particularly due to its practicality and efficacy in identifying malnutrition in emergency settings. However, our study builds upon this by using MUAC in a context of intensified conflict, reflecting the escalating malnutrition risks and worsening living conditions. This approach adds depth to our findings, as it captures the nutritional impact under the unique, severe conditions currently affecting Gaza’s population.

Furthermore, to enhance clarity, our study specifically includes children aged 6 months to 5 years. This age specification is critical, as it has implications for the prevalence of malnutrition, and underscores the acute vulnerability of this group to the crisis conditions in the Gaza Strip.

Additionally, there is a need for more detailed and current data to understand the extent of this issue and to identify specific socio-demographic factors that contribute to the prevalence of SAM and MAM. Such information is crucial for designing effective interventions to combat malnutrition in this context.

This study aims to fill this gap by assessing the occurrence of SAM and MAM among children under five in the Gaza Strip during a period of conflict. Specifically, this research seeks to address the following questions: What is the prevalence of SAM and MAM among children under five in conflict-affected areas of the Gaza Strip? What socio-demographic factors, such as age, sex, household income, parental education, and displacement status, are associated with higher rates of SAM and MAM? Are there regional differences in the prevalence of SAM and MAM within the Gaza Strip?

In light of these research questions, we hypothesize that the prevalence of both SAM and MAM will be higher in conflict-affected areas of the Gaza Strip compared to global averages for similar populations. We also hypothesize that socio-demographic factors, including lower household income, lower parental education levels, and displacement status, will be significantly associated with higher rates of SAM and MAM. Furthermore, we anticipate that there will be regional differences in malnutrition prevalence within the Gaza Strip, with certain areas exhibiting disproportionately higher rates due to more severe conflict-related disruptions.

Understanding the socio-demographic determinants of malnutrition is essential for developing targeted nutritional programs and interventions (19). By identifying the most affected groups and regions, resources can be allocated more effectively, ensuring that those in greatest need receive timely and adequate support. This study, therefore, represents a critical step towards addressing the malnutrition crisis in the Gaza Strip and improving the health outcomes of its youngest residents.

## Materials and methods

### Study design and setting

The study was conducted in the Gaza Strip between February 2 and June 18, 2024. The Gaza Strip is a densely populated area with significant socio-economic challenges exacerbated by ongoing conflict (20). The Gaza Strip is a 365 square kilometer coastal territory bordered by Israel to the north and east, Egypt to the southwest, and the Mediterranean Sea to the west (21). This addition helps to contextualize the unique geographic and political challenges that contribute to the region’s current humanitarian crisis. The Gaza Strip is divided into three main regions: North Gaza, Deir al-Balah (Middle Area), and South Gaza.

### Study population

The study targeted children under 5 years of age (aged 6 to 60 months), residing in the Gaza Strip.

### Eligibility criteria

#### Inclusion criteria

- Children aged between 6 months to 5 years.
- Residing in the Gaza Strip during the study period.
- Available for participation in the study and consent provided by guardians or caretakers.

#### Exclusion criteria

- Children outside the specified age range (under 6 months or over 5 years).
- Children residing outside the Gaza Strip during the study period.
- Inability or refusal to participate in the study by guardians or caretakers.

### Sampling method

A stratified random sampling method was employed to ensure representation from different living conditions within each region. A total of 1,200 children were screened, with 400 children selected from each of the three regions. The sample size was determined based on prevalence estimates from previous studies and adjusted for expected non-response.

### Data collection procedures

Data were collected through direct interviews with parents or guardians and physical examinations of the children. Trained healthcare professionals conducted the interviews and measurements. The questionnaire used in the study was developed based on standardized tools employed in previous nutritional and socio-demographic assessments, and it was tailored to the local context through expert consultations. To ensure its validity, the questionnaire was piloted on a small sample of 50 families before the main study began, and adjustments were made to improve clarity and cultural relevance. Reliability was ensured by performing inter-rater reliability checks, and the Cronbach’s alpha was calculated to assess internal consistency, achieving a score of 0.82, which indicates acceptable reliability.

Standardized questionnaires were used to gather socio-demographic information, and MUAC measurements were taken using standardized techniques (22).

### Nutritional assessment

The MUAC was used to assess the nutritional status of the children. MUAC measurements were taken at the midpoint of the left upper arm using a non-stretchable tape. Nutritional status was categorized as Normal, SAM, or MAM, based on established cut-off points (SAM, defined by a MUAC <115 mm) and (MAM, defined by a MUAC ≥115 mm) (23, 24). MUAC is particularly valuable in conflict settings like Gaza due to its simplicity, ease of use, and effectiveness in identifying acute malnutrition in resource-limited environments where more comprehensive assessments may not be feasible (15).

### Variables and measurements

*Gender:* Categorized as male or female.

*Age:* Recorded in months and categorized into two groups: six to less than 24 months, and 24 to 60 months.

*Family size:* The total number of family members living in the household.

*Living conditions:* Categorized as living in a house, school, or tent.

*Nutritional status:* Assessed using MUAC measurements and classified into three categories: Normal, SAM, and MAM.

### Data management

Data were recorded on paper forms and subsequently entered into a secure electronic database. To further ensure data reliability, double data entry was conducted by two independent personnel, and regular consistency checks were performed. Missing data were handled using appropriate statistical methods such as multiple imputation. All data were stored in a password-protected database to maintain confidentiality, with access restricted to authorized research personnel.

### Statistical analysis

Data were analyzed using IBM-SPSS, version 26. Descriptive statistics summarized the characteristics of the screened cases, including percentages and frequencies for categorical variables. Inferential statistics, including Chi-square tests, Fisher’s exact tests, and logistic regression analyses, were conducted to identify associations between socio-demographic characteristics and malnutrition status. Missing data were handled using appropriate statistical methods, and all tests were performed at a 0.05 significance level.

### Ethical considerations

The study protocol was reviewed and approved by the ethical review board at Al-Israa University. Informed consent was obtained from the parents or guardians of all participating children. In addition to institutional approval, potential ethical risks, such as distress caused to the children during physical examinations, were considered, and procedures were put in place to minimize discomfort. Trained personnel were present to ensure children’s well-being throughout the assessment. Moreover, the confidentiality of participants was protected not only through secure data storage but also by anonymizing all personal identifiers before data analysis. The study was conducted in accordance with the ethical standards of the Helsinki Declaration.

## Results

### Socio-demographic characteristics of the screened cases

A total of 1,200 children under 5 years of age were screened for nutritional status across three governorates in the Gaza Strip (North Gaza, Middle Zone, and South Gaza), with 400 children from each governorate. The gender distribution among the screened children was balanced across the regions, with 605 (50.8%) males and 595 (49.2%) females. The age distribution of the children screened showed that 48.4% (*n* = 581) fall within the range of 6 months to <24 months category, and the remaining 51.6% (*n* = 619) fall within the range of 24–60 months (2–5 years) group. Most families have between 4 to 9 members, with an overall percentage of 82.4%. North Gaza has a slightly higher percentage of households with more than 9 members (10.5%), compared to Middle Zone and South Gaza. The South Gaza has the highest proportion of smaller families (1–3 members) at 17.5%, followed by Middle Zone (15.0%).

Overall, 58.3% of children live in schools (used as shelters), 26.6% live in houses, and 15.1% reside in tents. North Gaza has the highest percentage of children living in schools (65.0%), whereas South Gaza has more children living in tents (18.8%) and houses (31.5%) compared to the other regions.

Nutritional status shows that 83.7% of children fall within the Normal range across all regions. The prevalence of SAM is highest in North Gaza and South Gaza (both at 6.8%) and slightly lower in the Middle Zone (6.5%). For MAM, the highest prevalence is in South Gaza (11.5%), with Middle Zone showing the lowest rate (7.3%) (Table 1).

**Table 1:** Socio-demographic characteristics of the screened cases (*n* = 1,200).

Variable	North Gaza	Middle Zone	South Gaza	Total
	*n* = 400 (%)	*n* = 400 (%)	*n* = 400 (%)	*n* = 1,200 (%)
Gender				
Male	207 (51.8)	200 (50.0)	198 (49.5)	605 (50.8)
Female	193 (48.2)	200 (50.0)	202 (50.5)	595 (49.2)
Age (Months)				
6 months- < 24 months	308 (77.0)	133 (33.2)	140 (35.0)	581 (48.4)
24–60 months (2–5 years)	92 (23.0)	267 (66.8)	260 (65.0)	619 (51.6)
Family members				
1–3	35 (8.8)	60 (15.0)	70 (17.5)	165 (13.8)
4–9	323 (80.8)	318 (79.5)	308 (77.0)	949 (82.4)
>9	42 (10.4)	22 (5.5)	22 (5.5)	86 (3.8)
Living place				
House	92 (23.0)	101 (25.2)	126 (31.5)	319 (26.6)
School	260 (65.0)	240 (60.0)	199 (49.7)	699 (58.3)
Tent	48 (12.0)	59 (14.8)	75 (18.8)	182 (15.1)
Nutrition status				
Normal	332 (82.9)	345 (86.2)	327 (81.7)	1,004 (83.7)
SAM	27 (6.8)	26 (6.5)	27 (6.8)	80 (6.7)
MAM	41 (10.3)	29 (7.3)	46 (11.5)	116 (9.6)

SAM, Severe Acute Malnutrition; MAM, Moderate Acute Malnutrition.

### Socio-demographic characteristics of malnutrition cases

Table 2 illustrates the prevalence of SAM and MAM across socio-demographic characteristics. Gender differences show that 6.1% of male children had SAM and 8.4% had MAM, while among females, SAM and MAM were observed in 7.3 and 10.9%, respectively, with no significant differences (*p* = 0.186). Age-related analysis reveals that children aged 6 to <24 months experienced the highest prevalence of SAM (11.0%) and MAM (17.9%), underscoring early childhood as a critical period for malnutrition. Family size showed no significant association with malnutrition, although children from households with 1–3 members had the highest prevalence of SAM at 8.5%; while children from households with 4–9 members had the highest prevalence of MAM at 10.1% (*p* = 0.875).

**Table 2:** Association between socio-demographic characteristics and malnutrition status.

Variables	Total (*N*) %	Normal (*N*) %	MAM (*N*) %	SAM (*N*) %	*p*-value
	*N* = 1,200 (100%)	*N* = 1,004 (83.7%)	*N* = 116 (9.6%)	*N* = 80 (6.7%)	
Gender					
Male	605 (50.8)	517 (85.5)	51.0 (8.4)	37.0 (6.1)	0.186
Female	595 (49.2)	487 (81.8)	65.0 (10.9)	43.0 (7.3)	
Age group (months)					
6 months- < 24 months	581 (48.4)	413 (71.1)	104 (17.9)	64.0 (11.0)	0.165
24–60 months (2–5 years)	619 (51.6)	591 (95.5)	12.0 (1.9)	16.0 (2.6)	
Family member					
1–3	165 (13.8)	135 (81.8)	16.0 (9.7)	14.0 (8.5)	0.875
4–9	949 (82.4)	790 (83.2)	96.0 (10.1)	63.0 (6.7)	
> 9	86 (3.8)	79.0 (81.9)	4.0 (4.7)	3.0 (3.4)	
Living place					
House	319 (26.6)	229 (71.8)	49.0 (15.4)	41.0 (12.8)	0.001
School	699 (58.2)	619 (88.6)	51.0 (7.3)	29.0 (4.1)	
Tent	182 (15.2)	156 (85.7)	16.0 (8.8)	10.0 (5.5)	
Region					
North-Gaza	400 (33.3)	332 (83.0)	41 (10.3)	27 (6.8)	0.001
Middle-Zone	400 (33.3)	345 (86.3)	29 (7.3)	26 (6.5)	
South-Gaza	400 (33.3)	327 (81.8)	46 (11.5)	27 (6.8)	

SAM, Severe Acute Malnutrition; MAM, Moderate Acute Malnutrition.

Living conditions significantly impacted malnutrition status, with children residing in houses exhibiting the highest rates 12.8% for SAM, and 15.4% for MAM, compared to those in schools or tents (*p* = 0.001). Regional differences were pronounced, the prevalence of SAM is highest in North Gaza and South Gaza (both at 6.8%) and slightly lower in the Middle Zone (6.5%). For MAM, the highest prevalence is in South Gaza (11.5%), with Middle Zone showing the lowest rate (7.3%) (*p* = 0.001).

### Association between living conditions, governorates, and malnutrition status

Table 3 displays the results of the logistic regression analysis examining the association between living conditions, governorates, and malnutrition status.

**Table 3:** Logistic regression analysis of malnutrition status and independent variables (*n* = 1,200).

Variable	MAM	MAM	SAM	SAM
	OR (95% CI)	*p*-value	OR (95% CI)	*p*-value
Living place				
Tent	**Ref**		**Ref**	
School	2.08 (1.14–3.80)	**0.016**	2.79 (1.35–5.74)	**0.005**
House	1.44 (1.13–1.84)	**0.003**	2.36 (1.39–3.99)	**0.001**
Governorate				
South-Gaza	**Ref**		**Ref**	
Middle-Zone	1.14 (0.539–2.44)	0.721	0.495 (0.149–1.66)	0.257
North-Gaza	10.51 (5.74–19.3)	**0.001**	13.2 (6.23–27.95)	**0.001**

Bold values means significant association. OR denotes Odds Ratio; Ref denotes Reference. SAM, Severe Acute Malnutrition; MAM, Moderate Acute Malnutrition.

For MAM cases, children living in schools, and houses had a higher likelihood of experiencing MAM (OR = 2.08; 95% CI = 1.14–3.80; *p* = 0.016), and (OR = 1.44; 95% CI = 1.13–1.84; *p* = 0.003), respectively.

For SAM cases, children living in schools, and houses also had a significantly higher likelihood of experiencing SAM (OR = 2.79; 95% CI = 1.35–5.74; *p* = 0.005), and (OR = 2.36; 95% CI = 1.39–3.99; *p* = 0.001), respectively. Unexpectedly, children residing in tents did not show a significantly increased risk of malnutrition compared to those in houses, suggesting that the temporary nature of tents may not be as detrimental as previously thought. Additionally, children in North Gaza were over 10 times more likely to suffer from 13 times more likely to experience SAM (OR = 13.2; 95% CI = 6.23–27.95; *p* = 0.001) and MAM (OR = 10.51; 95% CI = 5.74–19.3; *p* = 0.001) compared to those in South Gaza. These results highlight significant associations between malnutrition status and living conditions, as well as regional disparities within the Gaza Strip, aligning with the study’s objective to identify critical risk factors for malnutrition.

## Discussion

This study provides a detailed assessment of malnutrition prevalence and associated factors among children under five in the Gaza Strip during a period of conflict. With an overall malnutrition prevalence of 16.6%, including 6.7% with SAM and 9.7% with MAM, the findings underscore the severe nutritional challenges faced by children in this conflict-affected region.

Table 2 shows that children in both houses and schools experience relatively high rates of SAM and MAM, reflecting widespread vulnerability across different shelter types. However, the logistic regression model in Table 3 indicates a significantly higher likelihood of SAM and MAM among children residing in houses and schools compared to those in tents. This discrepancy suggests that additional confounding factors may influence the risk for malnutrition in children residing in houses and schools. For example, children in schools may be exposed to more compounded vulnerabilities, such as higher population density, less consistent access to clean water, and possibly greater psychosocial stressors, which might not be fully apparent from prevalence rates alone. Regarding, the low proportion of children with MAM and SAM who living tents, could be explained by that they are exposed to more nutrition intervention programs which usually focused on the family residing in tents compared to those who living in houses and schools. Moreover, it is may reflect the focus of nutrition programs targeting displaced families in these temporary shelters (25). This targeted intervention approach likely reduces malnutrition prevalence in tented populations, a factor that could also influence the relative odds observed in the adjusted model.

These findings highlight the need for comprehensive, context-specific nutritional interventions that address the unique vulnerabilities of children residing in houses, schools and other overcrowded shelters, as well as the importance of continued support for displaced populations in tents.

The prevalence rates observed in this study, although similar to those reported in other conflict zones (26, 27), must be understood in the context of the current unprecedented humanitarian crisis in Gaza. Recent estimates indicate that all 2.2 million people in the Gaza Strip are facing acute food insecurity, contributing to catastrophic conditions (12). The ongoing blockade, coupled with the intensification of military operations, has caused extreme damage to infrastructure and vital services, severely disrupting access to food, clean water, and healthcare (28–30). The malnutrition rates observed in this study should not merely be compared to other conflict zones but should reflect the unique and dire circumstances facing Gaza today, where the severity of food insecurity and deprivation surpasses many other crisis settings.

The study identified several key socio-demographic factors associated with malnutrition, including living conditions and regional disparities. Children residing in shelters, such as schools, were found to have a significantly higher likelihood of experiencing SAM (OR = 2.79) and MAM (OR = 2.08) compared to those living in tents, while children residing in houses, were found to have a significantly higher likelihood of experiencing SAM (OR = 2.36) and MAM (OR = 1.44) compared to those living in tents. This finding reflects the heightened vulnerability of displaced populations in Gaza, where overcrowding, lack of sanitation, and restricted access to food are more severe than in other conflict settings. In houses, lack of nutrition intervention programs and restricted access to food could be the main factors of the increased malnutrition prevalence in children. The significant regional differences in malnutrition rates, with children in North Gaza being at a higher risk for both SAM (OR = 13.2) and MAM (OR = 10.51) compared to those in South Gaza, also highlight localized challenges. These may be attributed to North Gaza’s more frequent exposure to military operations and blockades, limiting access to essential services (31).

Interestingly, children residing in tents showed a lower prevalence of SAM and MAM compared to those in other living conditions. This result may partly reflect the high concentration of humanitarian and nutritional assistance programs targeting children displaced in tent settlements. Many relief organizations prioritize tent camps for intervention efforts, given the perceived vulnerability of those living in temporary and often more exposed environments (25). However, it is essential to consider the possibility of selection bias, as children experiencing severe malnutrition in tents may be less likely to participate in studies due to limited mobility, lack of access to survey sites, or health conditions that preclude attendance. Furthermore, this result should be interpreted with caution, as it does not necessarily suggest that displacement in tents offers any nutritional advantage but rather that these children may be receiving more intensive support relative to other displaced populations.

While this study provides valuable insights, it is crucial to address several limitations that may influence the interpretation of the results. First, the cross-sectional design limits the ability to establish causal relationships between socio-demographic factors and malnutrition outcomes. Additionally, the questionnaire used for data collection, while comprehensive, lacked formal validation, raising concerns about the reliability of the data. The reliance on self-reported information from caregivers introduces the potential for recall bias, particularly concerning socio-economic conditions and healthcare access. Furthermore, missing data, though minimal, could have impacted the analysis, particularly in regions where data collection was hindered by conflict-related barriers. Another limitation is potential selection bias where those who were at highest risk of malnutrition were not included in the analysis. To overcome these barriers, the study data was collected by well-trained data collectors and using standard questionnaire and methods; pilot study was conducted before data collection; to further ensure data reliability, double data entry was conducted by two independent personnel, and regular consistency checks were performed; and missing data were handled using appropriate statistical methods.

The findings of this study have important implications for public health interventions in conflict zones, particularly in the Gaza Strip. The high prevalence of SAM and MAM among children underscores the urgent need for targeted nutritional interventions. These interventions should prioritize improving living conditions in shelters, ensuring food security, and enhancing access to healthcare services, particularly in the most vulnerable regions such as North Gaza. Furthermore, region-specific approaches are necessary to address the varying levels of risk across the governorates.

Future research should focus on longitudinal studies to better understand the long-term impact of conflict on child malnutrition and health outcomes. Additionally, studies that formally validate data collection tools and explore the mechanisms driving regional disparities in malnutrition would enhance the evidence base for more effective interventions. Understanding these factors is critical for designing targeted and context-specific strategies that can mitigate the impact of conflict on child nutrition.

## Conclusion

This study provides a critical examination of the prevalence and socio-demographic factors associated with SAM and MAM among children under 5 years old in the Gaza Strip during a period of conflict. The findings reveal alarmingly high rates of malnutrition, particularly among children residing in houses, shelters such as schools, and those living in the North Gaza governorate.

The results underscore the significant impact of living conditions and regional disparities on nutritional status. Children living in houses and schools were found to have a substantially higher likelihood of experiencing both SAM and MAM compared to those living in tents, highlighting the detrimental effects of displacement and inadequate houses and shelter conditions. Furthermore, the stark differences in malnutrition rates between governorates point to the urgent need for region-specific interventions.

The study’s findings emphasize the importance of targeted nutritional interventions and support for the most vulnerable groups. Humanitarian efforts should prioritize improving living conditions in houses and shelters and addressing the unique challenges faced by families in North Gaza. Additionally, there is a critical need for continuous monitoring and assessment of the nutritional status of children in conflict zones to enable timely and effective responses.

*Future research should focus on longitudinal studies* that track the nutritional status of children in conflict-affected regions over time, to better understand the long-term impacts of displacement and conflict on malnutrition. Investigating the interplay between socio-demographic factors such as household size, parental education, and income levels with child nutrition outcomes would also provide deeper insights into the determinants of malnutrition. Furthermore, *studies exploring the effectiveness of various interventions*—such as fortified food distribution, nutritional supplementation programs, and improvements to shelter infrastructure—are essential for developing evidence-based strategies to reduce malnutrition in conflict zones.

Policy recommendations should prioritize the following key areas:

1. Ensuring that displaced families living in houses, schools and other temporary shelters have access to adequate nutrition, clean water, and sanitation is critical for reducing malnutrition rates among children.
2. Tailoring nutritional aid and health services to address the distinct needs of different regions, particularly in North Gaza, where the highest malnutrition rates were observed, is essential for effectively combating SAM and MAM.
3. Long-term strategies should focus on rebuilding healthcare infrastructure, promoting food security, and ensuring stable, secure living environments. This can help prevent malnutrition and address its root causes.
4. Government authorities, international organizations, and community stakeholders must work together to implement multi-sectoral approaches that combine immediate nutritional aid with sustainable development efforts.

Addressing malnutrition in conflict-affected areas like the Gaza Strip requires a multifaceted approach. This includes not only immediate nutritional interventions but also long-term strategies to rebuild and strengthen healthcare systems, ensure food security, and provide stable living conditions. By fostering collaboration between local authorities, international organizations, and the community, stakeholders can mitigate the impact of conflict on children’s health and well-being.

## Data Availability

The datasets presented in this study can be found in online repositories. The names of the repository/repositories and accession number(s) can be found in the article/supplementary material.
